# Impact of Specific Bowel Symptoms on Quality of Life in Patients with Midgut Neuroendocrine Tumours

**DOI:** 10.1007/s00268-021-06146-9

**Published:** 2021-05-09

**Authors:** Håkan Ohlsson, Gideon Wahlberg, Marlene Malmström, Rita Gustafsson, Anna Sundlöv, Erik Nordenström, Martin Almquist

**Affiliations:** 1 Department of Surgery, Ystad Hospital, VO Kirurgi, Lasarettet i Ystad, Kristianstadvägen 3, 271 33 Ystad, Sweden; 2 Institution of Clinical Sciences, Lund University, Lund, Sweden; 3 Department of Surgery, Skåne University Hospital, Lund, Sweden; 4 Department of Health Sciences, Lund University, Lund, Sweden; 5 Department of Oncology, Skåne University Hospital, Lund, Sweden; 6 Endocrine-Sarcoma Unit, Department of Surgery, Skåne University Hospital, Lund, Sweden

## Abstract

**Introduction:**

Patients with midgut neuroendocrine tumours (NETs) suffer from decreased health-related quality of life (HRQoL), in large part due to bowel symptoms. However, it is unknown which bowel symptoms affect HRQoL the most. An enhanced understanding of this is essential to better focus treatment on this aspect of the disease. This study aimed to determine which bowel symptoms affect HRQoL the most in patients with midgut NETs.

**Methods:**

Consenting patients with midgut NET completed the Memorial Sloan Kettering Bowel Function Instrument and the HRQoL questionnaire (EORTC QLQ-C30). The correlation between bowel symptoms and HRQoL was analysed using multiple linear regression, adjusting for age, Charlson Comorbidity Index score, presence of metastatic disease, chromogranin A, and BMI yielding ß-coefficients with 95% confidence intervals.

**Results:**

Totally, 119 patients with midgut NET completed the questionnaires and were included in the study. Loose stool and bowel frequency ≥ 3/day were the most common bowel symptoms, reported by 47% and 56% of patients, respectively. However, sensitivity to certain types of food and beverages, a feeling of incomplete emptying of the bowel, and soiling were the symptoms most strongly correlated with decreased HRQoL, especially within domains concerning role and social function, with ß-coefficients for the strongest correlated symptoms of 15.0 and 14.6, respectively.

**Discussion:**

While symptoms concerning stool consistency and frequency are common in patients with midgut NET, our study suggests that other, more socially stigmatising symptoms affect patients’ HRQoL more. Our findings could help caregivers understand patients’ perceptions of the disease and provide avenues for more directed therapies.

**Supplementary Information:**

The online version contains supplementary material available at 10.1007/s00268-021-06146-9.

## Introduction

Following improved treatment during the last decades, survival of patients with midgut neuroendocrine tumours (NET) has improved markedly [1]. Consequently, with improved survival, an increased focus has been placed on the patients’ self-experienced burden of the disease, i.e. health-related quality of life (HRQoL) [2, 3]. HRQoL is lower in patients with NET compared to the general population [4]. Causes of impaired HRQoL in patients with NET include symptoms such as fatigue, diarrhoea, and flushing [5, 6]. Unfortunately, as the disease progresses, several of these symptoms often worsen, despite maximal symptomatic treatment [7] leading to chronically impaired HRQoL.

Bowel symptoms are frequent in patients with midgut NET and are often referred to non-specifically as “diarrhoea”. Most previous studies on HRQoL in patients with NET have focused on the number of stool movements [6], but this aspect of bowel symptomatology might not in itself adequately portray the patients’ bowel symptom burden. Symptoms such as urgency to pass stool, leakage, and sensitivity to certain food or beverages might also contribute to impaired HRQoL. In patients with colorectal cancer, such bowel symptoms have been explicitly evaluated [8, 9] and found to decrease HRQoL. To our knowledge, only one smaller (*n* = 35), interview-based, qualitative study with this aim has been conducted on patients with NET [10]. This study indicated that symptoms related to bowel movement frequency and urgency had the greatest impact on patients HRQoL, especially within social, occupational, physical, and emotional domains. To further improve patients’ quality of life, a deeper understanding of which symptoms contribute the most to impaired HRQoL is imperative.

Our aim, therefore, was to study the relationship between specific bowel symptoms and HRQoL to understand which bowel symptoms explain the variance of HRQoL the most in patients with midgut NET. Another aim was to compare their HRQoL to that of the general population.

## Methods

### Patients

Patients alive on 1 September 2019 in the southern hospital region of Sweden and whose histopathological diagnosis of well-differentiated (G1-G2) NET with origin in the gastrointestinal tract had been established between 1 January 2000 and 31 December 2018 were eligible for inclusion.

The following exclusion criteria were used:

- Non-metastasised neuroendocrine tumours (NET) that only required endoscopic resection
- Appendiceal NET where appendectomy sufficed as the only treatment
- NET found incidentally during resection of another cancer
- Synchronous inoperable colorectal cancer
- Synchronous inflammatory bowel disease

Patients were identified by searching for gastrointestinal NET codes according to the Systematized Nomenclature of Medicine (SNOMED) system (see supplement for details) in the pathological database of the Skåne healthcare region. Eligible patients were invited by regular mail. Patients were sent questionnaires, as described below, and an informed consent form. If a patient did not reply within one month, one reminder letter was sent to the patient. Patients who replied that they did not wish to participate, and patients who did not reply within two months after one reminder, were considered non-responders. To minimise heterogeneity in the cohort and since bowel symptoms are most common from tumours with origin in the middle GI-tract, only patients with origin from small intestine, right colon, or appendix, i.e. the group previously denoted midgut NET, were included in the subsequent analysis. The complete cohort will be used for further studies on HRQoL in patients with GEP-NET.

### Questionnaires

Since, to our knowledge, there is no bowel symptom instrument specifically designed and validated for patients with NET, we searched the literature for instruments used in patients with similar bowel symptoms. The Memorial Sloan Kettering Cancer Center Bowel Function Instrument (MSKCC-BFI) [11] consists of 18 items enquiring broadly and specifically into a range of bowel symptoms. This instrument has been validated for patients who have undergone rectal cancer surgery. The MSKCC-BFI uses plain English which, for this study, the authors translated into Swedish. Items in the MSKCC-BFI enquire how often symptoms occur: never, rarely, often, or always, except for the first item, which asks how many bowel movements a patient has per day. Answers to this item were transformed into quintiles, and answers from items 4, 5, 7, 11, and 12 were inverted, so that lower scores reflect more symptoms for all items, as per the reference manual [11].

Patients’ HRQoL was evaluated with the cancer-specific, generic European Organisation for Research and Treatment of Cancer questionnaire EORTC QLQ-C30 [12]. This 30-item instrument is used to construct one Global Quality of Life scale (QL2) and five function scales: Physical Functioning (PF2), Role Functioning (RF2), Emotional Functioning (EF), Cognitive Functioning (CF), and Social Functioning (SF). These six scales were considered outcome variables and their values calculated and transformed into linear scales with 100 representing optimal function or high quality of life, according to the EORTC reference manual [13]. Answers from the diarrhoea symptom scale (DI) were reversed so that the value 100 indicates a severe symptom burden and 0 no symptoms.

#### Medical records/medical information

Information on previous disease, primary tumour site, presence of distant metastasis/residual tumour, Ki67 index at primary histopathological diagnosis, tumour grade, previous tumour surgeries, treatment with somatostatin analogue (SSA), treatment with anti-diarrhoeal agents or laxatives, levels of 24-h urine 5-hydroxyindoleacetic acid (U 5-HIAA), levels of serum chromogranin A, years since diagnosis, and previous or ongoing peptide-related radionuclide therapy (PRRT) or chemotherapy was obtained from patients’ electronic medical records.

### Statistical analysis

#### Comparison with the general population

To allow comparison with the general population, the difference between values of QL2, function scales, and DI in our cohort and corresponding values for the general Swedish population aged 60–79 years [14] was calculated, yielding delta values. A one-sided t-test was used to investigate whether the difference was significant compared to the reference values mentioned above. Delta values 5–10 are generally considered small, 10–20 moderate, and > 20 large in a clinical context [15].

#### Missing data

Missing values from the MSKCC-BFI were imputed using multiple imputation [16] with predictive mean matching [17], k-nearest neighbour (knn) = 10, and 20 imputations using a fixed random seed number. Missing values for U 5-HIAA and Chromogranin A (CgA) were recoded into within reference range, above reference range, or missing. The outcome variables HRQoL function and global scales were not imputed; patients without information on specific outcome variables were left out of the analysis.

#### Regression analysis

Multiple linear regression analysis using each function scale and the global QL2 as outcome variables was performed to investigate the relationship between each symptom item as reported in the MSKCC-BFI and HRQoL. Item 18, “Have you had to change daily activities due to bowel symptoms?” was omitted, since this item does not strictly enquire about symptoms, but about how symptoms affect daily activities.

The regression model was adjusted for patient and disease-related factors. Choice of inclusion of covariates was based on a combination of biological and statistical reasoning: Sex, age, Charlson Comorbidity Index score (Charlson CMI), U 5-HIAA, CgA, distant metastasis, any residual intra-abdominal tumour on radiology, SSA treatment, BMI, years since diagnosis, and type of tumour surgery were all included in the model based on a biological presumption that these variables are important for HRQoL. Of these, only variables with *p* < 0.1 in any of the models were retained in the main analysis.

In the first linear regression model, each item of the MSKCC-BFI was investigated separately; in the second model, all items of the MSKCC-BFI were included simultaneously.

## Results

Between 1 January 2000 and 31 December 2018, 806 unique patients were registered with GI-tract NET diagnosis codes. Of these, 561 patients were excluded following the predefined exclusion criteria. Between 2 September 2019 and 27 September 2019, the remaining 245 patients were invited by regular mail to participate in the study. Of these, three declined to participate, and 10 had to be excluded due to unknown address or emigration. A further 67 patients did not respond. The remaining 165 patients returned the completed instruments and signed the consent forms, resulting in a response rate of 67%. In this study, to minimise heterogeneity, only the 119 patients with small intestine, right colon, or appendix primary site were included.

### Patient characteristics

Mean (standard deviation, s.d.) age of the patients was 70.4 (10.7) years; 61% were men. Mean (s.d.) time since diagnosis was 6.4 (3.7) years. A total of 71 patients (60%) had metastatic disease at the time of the study, and 59 of them were receiving SSA treatment. A further 11 patients without metastatic disease were also receiving SSA treatment, giving a total of 70 patients (59%) receiving SSA treatment. Of the 51 patients (43%) with u-5HIAA levels above upper limit of normal (ULN), 16 patients had very high levels (> 10 × ULN, 300 µmol/d), indicating severe endocrinopathy; 112 patients had small intestine or right colon origin; 7 patients had appendix origin. Some 67 patients had undergone small bowel resection; 58 patients had undergone a right-sided resection. In 2 patients with small intestinal NET metastatic to the pancreas, pancreatic resection was performed in addition to bowel surgery. Resection of bowel was not associated with higher scores of the EORTC-GI.NET21 symptom subscale diarrhea (DI). For more details about treatment and patient factors, see Tables 1 and 2.

**Table 1:** Cohort characteristics

	Mean (s.d.)	Reference values general pop 60–79 years	Delta	*p* value for *t*-test	Median (i.q.r.)	n (%) with score < 50	*n*
Age	70.4 (10.7)				73 (64–78)		119
Charlson Comorbidity Index	3.9 (2.5)				3 (2–5)		119
Weight	76.4 (17.5)				75(65–86)		118
Height	171.7 (18.1)				173 (166–179)		118
BMI	25.4 (5.3)				24.8 (21.9–27.9)		118
Years since diagnosis	6.4 (3.7)				5.6 (3.5–8.8)		119
QL2 (0–100)	72.9 (20.9)	76.7	3.8	0.027	75 (58.3–83.3)	14 (11.8)	118
PF2 (0–100)	82.6 (20.1)	86.1	3.5	0.031	86.7 (73.3–100)	11 (9.2)	119
RF2 (0–100)	81.5 (28.4)	87.8	6.3	0.009	100 (66.7–100)	17 (14.3)	119
EF (0–100)	81.8 (22.3)	87.5	5.7	0.003	91.7 (66.7–100)	12 (10.0)	118
CF (0–100)	84.8 (20.1)	88.0	3.2	0.05	91.7 (83.3–100)	6 (5.0)	118
SF (0–100)	80.6 (26.1)	91.3	10.7	< 0.001	100 (66.7–100)	13 (10.9)	118
DI (0–100)	35.9 (35.4)	5.4	30.5	< 0.001	33.3 (0–66.7)	38* (31.9*)	118
	Yes	No	Missing				
	*n* (%)	*n* (%)	*n* (%)				
Chromogranin A ≥ 2 nmol/L	63 (52.9)	51 (43.9)	5 (4.2)				
Urine 5-HIAA ≥ 30 µmol/d	51 (42.9)	62 (52.1)	6 (5.0)				
Male sex	73 (61.3)	46 (38.7)	–				
Metastatic disease	71 (59.7)	48 (40.3)	–				
Grade	Grade 1 (Ki67 0–2%)	Grade 2 (Ki67 3–20%)	Missing				
	n (%)	n (%)	n (%)				
	85 (72.0)	31 (26.3)	3 (2.5)				
	Ongoing	Previous	None				
	n (%)	n (%)	n (%)				
Chemotherapy	0 (0)	7 (5.9)	112 (94.1)				
PRRT	5 (4.2)	14 (11.8)	100 (84.0)				
SSA treatment	70 (58.8)		49 (41.2)				
Creon treatment	17 (14.3)		102 (85.7)				
Cholestyramine treatment	5 (4.2)		114 (95.8)				
Antidiarrheal medication	27 (22.7)		92 (77.3)				
Use of laxatives	6 (5.0)		113 (95.0)				
Telotristat treatment	3 (2.5)		116 (97.5)				

^†^Age-adjusted reference values from Derogar et al.

^*^For symptom scales, higher score indicates more severe symptoms

*QL2* Global Quality of Life, *PF2* Physical Function, *RF2* Role Function, *EF* Emotional Function, *CF* Cognitive Function, *SF* Social Function. *5-HIAA* 5-Hydroxyindoleacetic acid

**Table 2:** Previous tumour surgery

Previous Tumour surgery:	yes (%)	mean DI-score	no	mean DI-score	p-value for t-test
Small bowel resection	67 (56.3)	34.3	52 (43.7)	37.9	0.59
Rightsided hemicolectomy or ileocecal resection	58 (49.6)	37.9	61 (50.4)	33.9	0.54
Both small bowel resection and rightsided resection	10 (8.4)	36.7	109 (91.6)	35.8	0.94
Other colorectal resection	3 (2.5)	66.7	116 (97.5)	35.1	0.13
Liver resection	4 (3.4)	–	115 (96.6)	–	–
Distal pancreatic resection	2 (1.7)	–	117 (98.3)	–	–
No surgery	3 (2.5)	–	116 (97.5)	–	–

Categories not mutually exclusive, i.e. some patients have undergone several types of procedures

### Health-related quality of life and bowel symptoms

The mean values for QL2 and the five function scales were all significantly (*p* < 0.05) below the reference values of the general population aged 60–79 years in Sweden. However, only SF were sufficiently low compared to the general population to be considered clinically relevant. The mean value for the symptom scale “Diarrhoea” (DI) was 35.9 (s.d. 35.4), which is significantly higher than the 5.4 (delta 30.5) mean of the age-adjusted general population. See Table 1 for details.

Table 3 displays the results of the MSKCC-BFI. The mean number of bowel movements per day was 2.7 (s.d. 1.7, range: 1–9); 67 patients (57%) had > 3 bowel movements per day. The four most frequent symptoms (often/always) reported were: loose stool, *n* = 55 (47%), not being able to wait 15 min when about to have a bowel movement, *n* = 40 (34%), presence of loose stool, i.e. diarrhoea, *n* = 37, (31%), and having another bowel movement within 15 min of the last one, *n* = 28 (24%).

**Table 3:** Bowel symptom prevalence

Item		Reverted				Total (*n*)
1	Over the last 4 weeks, how many bowel movements do you generally have in 24 h?		mean 2.7 times/day (s.d. 1.7), range 1–9			115
			Always/most of the time (> 1/week) n (%)	Sometimes (1/week) n (%)	Rarely/never (< 1/week) n (%)	
2	Do certain solid foods increase the number of bowel movements in a day?		19 (16.0)	40 (33.6)	60 (50.4)	119
3	Do certain liquids that you drink increase the number of bowel movements in a day?		14 (11.8)	17 (14.3)	88 (74.0)	119
4	Do you feel like you have totally emptied your bowels after a bowel movement?	yes	79 (67.0)	24 (20.3)	15 (12.7)	118
5	Do you get to the toilet on time?	yes	105 (89.0)	6 (5.1)	7 (5.9)	118
6	Do you have another bowel movement within 15 min of your last bowel movement?		28 (23.5)	28 (23.53)	63 (52.9)	119
7	Do you know the difference between having to pass gas (air) and needing to have a bowel movement?	yes	80 (67.8)	18 (15.3)	20 (17.0)	118
8	Have you used medicines to decrease the number of bowel movements?		25 (21.2)	11 (9.3)	82 (69.5)	118
9	Have you had diarrhea (no form, watery stool)?		37 (31.4)	29 (24.6)	52 (44.1)	118
10	Have you had loose stool (slight form, but mushy)?		55 (46.6)	38 (32.2)	25 (21.2)	118
11	Have you been able to wait 15 minutes to get to the toilet when you feel like you are going to have a bowel movement?	yes	47 (39.8)	31 (26.3)	40 (33.9)	118
12	Have you been able to control the passage of gas (air)?	yes	68 (58.1)	33 (28.2)	16 (13.7)	117
13	Have you limited the types of solid foods you eat to control your bowel movements?		16 (13.5)	18 (15.1)	85 (71.4)	119
14	Have you limited the types of liquids you drink to control you bowel movements?		6 (5.0)	14 (11.8)	99 (83.2)	119
15	Have you had soilage (leakage of stool) of your undergarments during the day?		11 (9.2)	21 (17.6)	87 (73.1)	119
16	Have you used a tissue, napkin, and/or pad in your undergarments during the day in case of stool leakage?		14 (11.8)	8 (6.7)	97 (81.5)	119
17	Have you had soilage (leakage of stool) of your undergarments when you go to bed?		2 (1.7)	7 (5.9)	110 (92.4)	119
18	Have you had to alter your activities because of your bowel function?		25 (21.2)	14 (11.9)	79 (67.0)	118

Adjusted multiple linear regression showed that age, Charlson CMI, presence of metastatic disease, chromogranin A above reference range, and BMI were independently associated with at least one of the outcome variables with significance level *p* < 0.1. Consequently, these were included in the subsequent multiple regression models.

Table 4 displays the results from the repeated, separate, multiple regression models investigating the association between bowel symptoms and QL2/function scales. All significant associations were positive, indicating lower quality of life with more symptoms. Symptoms concerning soiling of undergarments, having to limit types of liquids or food, feeling of incomplete emptying of bowel had the most impact on both QL2 and the five function scales. For the subscales Role Function and Social Function, the ß-coefficients were generally higher than for the other subscales, e.g. 13.0, 95% CI: 8.3–17.6 for RF2 vs item 14 (not getting to the toilet on time).

**Table 4:** Multiple linear regression, symptoms analysed in separate models. Adjusted for age, Charlson Comorbidity Index, Chromogranin A level above reference range, metastatic disease, BMI

		QL2	PF2	RF2	EF	CF	SF
		ß-coefficient	ß-coefficient	ß-coefficient	ß-coefficient	ß-coefficient	ß-coefficient
Item	Question	(95% CI)	(95% CI)	(95% CI)	(95% CI)	(95% CI)	(95% CI)
1	Over the last 4 weeks, how many bowel movements do you generally have in 24 h?	4.18	3.28	4.39	1.31	1.61	3.75
		(1.71–6.66)	(1.00–5.55)	(0.97–7.82)	( 1.50–4.12)	( 0.90–4.14)	(0.54–6.96)
2	Do certain solid foods increase the number of bowel movements in a day?	3.51	5.77	6.54	1.64	2.32	5.91
		( 0.01–7.05)	(2.57–8.96)	(1.69–11.39)	( 2.28–5.58)	( 1.20–5.85)	(1.53–10.30)
3	Do certain liquids that you drink increase the number of bowel movements in a day?	3.48	3.84	4.50	1.72	3.15	4.18
		(0.11–6.86)	(0.69–7.01)	( 0.22–9.23)	( 2.03–5.48)	( 0.19–6.50)	( 0.07–8.43)
4	Do you feel like you have totally emptied your bowels after a bowel movement?	6.34	7.14	9.95	7.62	3.83	8.76
		(2.97–9.71)	(4.05–10.24)	(5.30–14.60)	(3.96–11.29)	(0.36–7.30)	(4.57–12.95)
5	Do you get to the toilet on time?	5.21	7.19	7.99	5.28	5.83	7.99
		(1.13–9.30)	(3.48–10.91)	(2.32–13.65)	(0.79–9.78)	(1.82–9.84)	(2.93–13.05)
6	Do you have another bowel movement within 15 min of your last bowel movement?	4.02	5.31	4.83	4.90	4.01	6.35
		(0.93–7.11)	(2.50–8.12)	(0.50–9.16)	(1.53–8.26)	(0.95–7.06)	(2.53–10.17)
7	Do you know the difference between having to pass gas (air) and needing to have a bowel movement?	2.72	1.94	3.44	2.19	1.98	3.76
		( 0.18–5.62)	( 0.81–4.70)	( 0.62–7.51)	( 1.02–5.40)	( 0.91–4.87)	(0.11–7.41)
8	Have you used medicines to decrease the number of bowel movements?	5.46	4.77	5.63	4.92	2.36	4.03
		(3.29–7.64)	(2.65–6.88)	(2.40–8.85)	(2.43–7.41)	(0.02–4.71)	(1.06–7.00)
9	Have you had diarrhoea (no form, watery stool)?	3.80	4.42	4.27	3.26	3.43	6.60
		(0.64–6.96)	(1.47–7.37)	( 0.21–8.76)	( 0.24–6.78)	(0.29–6.57)	(2.72–10.48)
10	Have you had loose stool (slight form, but mushy)?	1.75	0.68	0.82	1.04	1.30	2.15
		( 1.61–5.12)	( 2.49–3.87)	( 3.89–5.54)	( 4.75–2.65)	( 4.63–2.02)	( 2.07–6.38)
11	Have you been able to wait 15 min to get to the toilet when you feel like you are going to have a bowel movement?	2.66	3.79	3.29	2.89	2.33	3.78
		(0.03–5.30)	(1.35–6.23)	( 0.43–7.02)	( 0.00–5.80)	( 0.29–4.95)	(0.48–7.08)
12	Have you been able to control the passage of gas (air)?	5.49	4.88	5.61	4.40	3.68	7.57
		(2.29–8.69)	(1.84–7.93)	(1.01–10.21)	(0.79–8.01)	(0.42–6.94)	(3.58–11.57)
13	Have you limited the types of solid foods you eat to control your bowel movements?	6.57	6.16	10.01	7.53	3.73	9.85
		(3.95–9.19)	(3.69–8.63)	(6.42–13.59)	(4.69–10.38)	(0.95–6.51)	(6.71–12.99)
14	Have you limited the types of liquids you drink to control you bowel movements?	7.79	8.56	12.96	9.91	5.24	11.00
		(4.35–11.2)	(5.42–11.7)	(8.32–17.59)	(6.25–13.56)	(1.67–8.81)	(6.77–15.22)
15	Have you had soilage (leakage of stool) of your undergarments during the day?	6.92	8.80	12.70	8.96	8.18	11.82
		(3.69–10.12)	(5.99–11.62)	(8.50–16.91)	(5.54–12.39)	(5.10–11.26)	(8.05–15.59)
16	Have you used a tissue, napkin, and/or pad in your undergarments during the day in case of stool leakage?	5.47	7.28	8.50	6.21	4.42	7.14
		(2.64–8.31)	(4.78–9.77)	(4.60–12.40)	(3.12–9.31)	(1.56–7.29)	(3.58–10.68)
17	Have you had soilage (leakage of stool) of your undergarments when you go to bed?	10.08	9.27	15.00	11.47	7.53	14.61
		(4.73–15.42)	(4.38–14.16)	(7.84–22.16)	(5.64–17.31)	(2.10–12.96)	(8.02–21.19)
18	Have you had to alter your activities because of your bowelfunction?	8.65	8.31	12.70	7.46	5.77	10.44
		(6.32–10.98)	(6.14–10.47)	(9.55–15.85)	(4.69–10.24)	(3.19–8.36)	(7.46–13.41)

*QL2* Global Quality of Life, *PF2 *Physical Function, *RF2* Role Function, *EF* Emotional Function, *CF* Cognitive Function, *SF* Social Function

Table 5 displays the results of the multiple linear regression, including adjusting variables and all items from the MSKCC-BFI except item 18 (step 2 of the main analysis). Only the symptoms “feeling that bowel has not fully emptied after bowel movement” (item 4), “having to limit food to control bowel movements” (item 13) and “soiling of undergarments during the day” (item 15) were statistically significantly associated with HRQoL. For the subscales Global Quality of Life (QL2) and Physical Function (PF2), no symptom was statistically significant.

**Table 5:** Multiple linear regression, symptoms analysed in same model. Adjusted for age, Charlson Comorbidity Index, Chromogranin A level above reference range, metastatic disease, BMI

		QL2	PF2	RF2	EF	CF	SF
		ß-coefficient	ß-coefficient	ß-coefficient	ß-coefficient	ß-coefficient	ß-coefficient
Item	Question	(95% CI)	(95% CI)	(95% CI)	(95% CI)	(95% CI)	(95% CI)
1	Over the last 4 weeks, how many bowel movements do you generally have in 24 h?	2.27	0.01	0.98	-2.32	-0.36	-1.74
		( 1.07–5.63)	( 2.73–2.76)	( 3.21–5.17)	( 5.96–1.30)	( 3.81–3.09)	( 5.67–2.18)
2	Do certain solid foods increase the number of bowel movements in a day?	1.14	3.18	1.45	2.25	0.30	0.65
		( 5.47–3.18)	( 0.51–6.88)	( 4.16–7.07)	( 6.83–2.31)	( 4.84–4.23)	( 4.34–5.65)
3	Do certain liquids that you drink increase the number of bowel movements in a day?	0.62	-0.95	0.99	0.35	1.57	1.25
		( 3.60–4.84)	( 4.63–2.73)	( 6.59–4.61)	( 4.81–4.11)	( 2.85–6.00)	( 6.13–3.62)
4	Do you feel like you have totally emptied your bowels after a bowel movement?	2.49	2.53	5.64*	3.85*	0.63	3.49
		( 1.70–6.69)	( 1.12–6.18)	(0.11–11.17)	( 0.59–8.31)	( 3.76–5.04)	( 1.36–8.35)
5	Do you get to the toilet on time?	0.92	4.28	3.13	0.36	1.29	0.92
		( 4.80–6.65)	( 0.73–9.29)	( 4.48–10.76)	( 6.44–5.71)	( 4.70–7.30)	( 5.70–7.55)
6	Do you have another bowel movement within 15 min of your last bowel movement?	2.14	0.31	3.31	0.03	0.54	0.50
		( 6.21–1.91)	( 3.12–3.76)	( 8.55–1.92)	( 4.24–4.30)	( 3.68–4.77)	( 5.17–4.17)
7	Do you know the difference between having to pass gas (air) and needing to have a bowel movement?	0.72	1.92	0.18	0.22	0.97	0.06
		( 4.19–2.73)	( 4.95–1.11)	( 4.41–4.79)	( 3.89–3.43)	( 4.60–2.66)	( 4.07–3.94)
8	Have you used medicines to decrease the number of bowel movements?	2.88	0.61	-0.19	0.53	0.93	3.60
		( 0.27–6.05)	( 2.19–3.42)	( 4.42–4.03)	( 2.82–3.89)	( 4.25–2.37)	( 7.27–0.06)
9	Have you had diarrhoea (no form, watery stool)?	3.73	0.24	3.50	0.42	2.53	1.94
		( 7.97–0.50)	( 3.94–3.46)	( 9.10–2.09)	( 4.89–4.05)	( 1.88–6.95)	( 2.93–6.82)
10	Have you had loose stool (slight form, but mushy)?	0.98	1.71	0.94	1.71	3.93*	0.01
		( 2.72–4.69)	( 4.92–1.49)	( 5.82–3.93)	( 5.62–2.18)	( 7.80–0.07)	( 4.26–4.22)
11	Have you been able to wait 15 min to get to the toilet when you feel like you are going to have a bowel movement?	0.19	0.24	0.50	0.61	0.58	0.12
		( 3.05–2.65)	( 2.28–2.77)	( 4.33–3.32)	( 2.40–3.63)	( 2.41–3.58)	( 3.17–3.42)
12	Have you been able to control the passage of gas (air)?	2.86	0.69	1.43	0.41	0.29	3.96
		( 1.18–6.91)	( 2.84–4.23)	( 3.92–6.79)	( 3.85–4.68)	( 4.52–3.93)	( 0.68–8.60)
13	Have you limited the types of solid foods you eat to control your bowel movements?	3.71	0.92	4.34	4.68*	1.03	6.47*
		( 0.04–7.46)	( 2.36–4.20)	( 0.64–9.32)	(0.71–8.65)	( 2.90–4.97)	(2.12–10.81)
14	Have you limited the types of liquids you drink to control you bowel movements?	0.45	2.68	5.37	3.05	0.74	3.15
		( 4.81–5.71)	( 1.91–7.28)	( 1.61–12.35)	( 2.48–8.60)	( 4.76–6.26)	( 2.92–9.22)
15	Have you had soilage (leakage of stool) of your undergarments during the day?	2.40	3.19	6.56*	4.10	7.19*	5.06
		( 2.25–7.05)	( 0.80–7.19)	(0.49–12.64)	( 0.80–9.02)	(2.31–12.08)	( 0.32–10.45)
16	Have you used a tissue, napkin, and/or pad in your undergarments during the day in case of stool leakage?	0.62	1.61	0.45	0.09	0.29	0.06
		( 4.65–3.41)	( 1.88–5.12)	( 5.78–4.87)	( 4.14–4.33)	( 4.50–3.91)	( 4.71–4.58)
17	Have you had soilage (leakage of stool) of your undergarments when you go to bed?	2.73	0.19	2.85	3.70	-1.39	4.07
		( 4.02–9.49)	( 5.66–6.06)	( 6.04–11.75)	( 3.46–10.87)	( 8.49–5.70)	( 3.75–11.90)
18	Have you had to alter your activities because of your bowel function?	–	–	–	–	–	–
		–	–	–	–	–	–

^*^ = *p* < 0.05. *QL2* Global Quality of Life, *PF2* Physical Function, *RF2* Role Function, *EF* Emotional Function, *CF* Cognitive Function, *SF* Social Function

## Discussion

In this study on 119 patients with midgut NET, HRQoL was significantly lower than in the general population. However, in line with previous studies [4, 6] the absolute difference was small. Furthermore, bowel symptoms were common; the most common was loose stool, *n* = 55 (47%), not being able to wait 15 min when about to have a bowel movement, *n* = 40 (34%), and having another bowel movement within 15 min of the last one, *n* = 28 (24%). All bowel symptoms affected HRQoL negatively, but the most common symptoms mentioned above did not have the most negative impact on HRQoL. Instead, symptoms of soiling, feeling that the bowel had not fully emptied after a bowel movement, and having to limit what to eat or drink, had the strongest negative effect on HRQoL.

To our knowledge, this has not been reported previously. A global, internet-based study in 2017 indicated that bowel symptoms are the next most common symptom in patients with NET, preceded only by fatigue/muscle weakness, and that the symptoms have a large impact on patients’ ability to lead their lives [18]. A smaller, qualitative study with 35 patients following a phase III trial of telotristat ethyl showed that an increased number of bowel movements and feelings of urgency associated with NET led to impaired emotional, social, physical, and occupational well-being [10]. The largest study so far to investigate the relationship between diarrhoea and HRQoL included 663 patients with NET [6] and showed a strong correlation between an increased number of bowel movements per day and worsened physical and social function. Our study complements these findings. The mechanisms for bowel symptoms are quite different in patients with midgut NET compared to patients having undergone surgery for colorectal cancer, suggesting that the findings from the latter group might not be applicable to the former. Nevertheless, the findings of our study correspond well to studies on bowel symptoms in patients with colorectal cancer [8, 9].

A potential explanation for the findings of this study is that while most patients with midgut NET experience frequent, loose stools, patients have learned to live with these symptoms. Conversely, symptoms concerning soiling and food intolerance can be expected to be associated with significant social stigma and thus might impair HRQoL more, especially within domains concerning social and role-functioning. Consequently, the results of the study indicate that to improve HRQoL in patients with midgut NET, in addition to reducing the number of bowel movements, the more troublesome symptoms of soiling, food intolerance, and feeling of incomplete bowel emptying should also be addressed. Hence, the present study points to potential future interventional trials.

Strengths of the present study include the population-based design, which reduces selection bias that can occur when inviting patients through patient organisations or other channels. The fact that different multiple regression models yielded similar results also strengthens the conclusions of the study.

One limitation of the study is that the MSKCC-BFI questionnaire has not been validated for patients with NET. To minimise the inferential risks from using a non-validated instrument, we chose not to base our regression models on the multi-item scales of the MSKCC-BFI. Instead, we included each question in the regression. This is in line with a previous study investigating bowel dysfunction after sigmoid resection due to colon cancer [8]. While there is a NET-specific instrument, the EORTC-QLQ GI.NET21 [19], we chose not to use it for the present study, since it does not inquire about bowel symptoms with sufficient detail. Another limitation is that only 14% of the patients in the cohort received Creon. Since the study was conducted, the use of Creon has become more prevalent in patients with SSA. This discrepancy might indicate that some patients in the cohort had untreated pancreas insufficiency. While this might have a small effect on the prevalence of bowel symptoms, it is unlikely to affect the relationship between bowel symptoms and HRQoL.

To conclude, this study confirms the high prevalence of bowel symptomatology for patients with a midgut NET diagnosis. It adds new, previously unknown relevant information about which bowel symptoms are the most frequent and which symptoms impact HRQoL the most. For patients with midgut NET, the go-to question for caregivers so far has concerned the number of bowel movements per day. While this variable is objective and easy to measure, this study indicates that other bowel symptoms might be more troublesome. Consequently, if the goal is increased HRQoL, this study suggests that caregivers should focus on other, more socially stigmatising symptoms.

## Supplementary Information

Below is the link to the electronic supplementary material.

Supplementary file1 (DOCX 12 kb)
