# Supplementary material for: Impact of Specific Bowel Symptoms on Quality of Life in Patients with Midgut Neuroendocrine Tumours
Source: World J Surg. 2021 May 9;45(9):2793–803. doi: 10.1007/s00268-021-06146-9 (PMC8321966; doi:10.1007/s00268-021-06146-9)
Supplement: Supplementary file 1 — Supplementary file1 (DOCX 12 kb) [file 268_2021_6146_MOESM1_ESM.docx]

Supplement:

SNOMED codes

T59000 pancreas NET

T63000 ventricle NET

T64000 duodenal NET

T65000 small intestine NET

T66000 appendiceal NET

T67000 colonic NET

T68000 rectal NET

M82403 NET G1 (carcinoid tumour)

M82493 NET G2

M81503 NET G1, G2 (non-functional)
